# Retrospective Observational Study of Treatment Patterns and Efficacy of onabotulinumtoxinA Therapy in Patients with Refractory Overactive Bladder in Clinical Practice

**DOI:** 10.3390/toxins15050338

**Published:** 2023-05-15

**Authors:** Kwang Jin Ko, Kyu-Sung Lee

**Affiliations:** 1 Department of Urology, Samsung Medical Center, Sungkyunkwan University School of Medicine, 81 Irwon-ro, Gangnam-gu, Seoul 06351, Republic of Korea; truelight8217@gmail.com; 2 Research Institute for Future Medicine Samsung Medical Center, Seoul 06351, Republic of Korea

**Keywords:** onabotulinumtoxinA, urinary incontinence, overactive bladder

## Abstract

This study aimed to evaluate the treatment patterns and long-term efficacy of onabotulinumtoxinA injections in a clinical setting. This single-center retrospective study was conducted on patients with refractory overactive bladder (OAB) aged 18 years or older who received onabotulinumtoxinA 100 IU administered between April 2012 and May 2022. The primary endpoint was the treatment pattern, including the retreatment rate and OAB medication prescription pattern. The duration and effectiveness of onabotulinumtoxinA treatment were analyzed using the overactive bladder symptom score and voiding diaries. A total of 216 patients were enrolled in this study, and the overall patient satisfaction rate was 55.1%. After the first injection, 19.9% received a second treatment, and 6.1% received three or more injections. The median duration until the second injection was 10.7 months. Among the patients, 51.4% resumed OAB medications after 2.96 months. The presence of urodynamic detrusor overactivity was observed only in female patients (odds ratio, 23.65; 95% CI, 1.84 to 304.40), which was associated with a good response. In contrast to clinical trials, the degree of improvement and retreatment rate did not meet expectations. Our findings provide valuable insights into the effectiveness of onabotulinumtoxinA injections in patients with refractory OAB symptoms in real-world practice.

## 1. Introduction

Overactive bladder (OAB) is a prevalent and debilitating condition that affects a significant portion of the global population, particularly women [1]. It is characterized by urgency, frequency, and nocturia, and in one-third of cases, it is accompanied by urgency urinary incontinence [2]. Initial management consists of lifestyle and behavioral modifications such as pelvic floor muscle exercises, bladder retraining, and fluid management. Pharmacological therapy is the second-line treatment for OAB, with antimuscarinic and beta-3 adrenergic agonist drugs being the most commonly used agents. Even with appropriate behavioral and pharmacological treatments, approximately 40% of patients do not achieve adequate symptom relief, which significantly affects their quality of life [3,4]. Several treatment options are available for patients with refractory symptoms [5,6]. One of the most promising options is onabotulinumtoxinA injections, which have been shown to significantly reduce urinary incontinence episodes and improve quality of life in clinical trials [7,8,9]. Sacral neuromodulation (SNM) is a more invasive procedure that involves the implantation of a device that delivers electrical impulses to the sacral nerve, which regulates bladder function [10].

OnabotulinumtoxinA injections have emerged as a minimally invasive treatment for patients with refractory OAB symptoms and have shown promising results in clinical trials [11,12]. OnabotulinumtoxinA is a neurotoxin produced by the bacterium Clostridium botulinum. It is known to block the release of acetylcholine, a neurotransmitter responsible for muscle contraction, by preventing the fusion of synaptic vesicles with the presynaptic membrane. The toxin binds to and cleaves SNAP-25, a protein involved in the release of acetylcholine, resulting in the inhibition of detrusor muscle contractions [13]. This leads to a decrease in bladder spasms, increased bladder capacity, and decreased urinary incontinence episodes in patients with refractory OAB. For patients with urgency urinary incontinence refractory to antimuscarinics, the European Association of Urology and American Urological Association guidelines state that 100 U of onabotulinumtoxinA can be offered [14]. However, few studies have examined the effectiveness of onabotulinumtoxinA injections in clinical practice [7,15,16,17], where patient characteristics and treatment modalities may differ from those in clinical trials. Therefore, we conducted a retrospective study to evaluate the treatment pattern and long-term efficacy of onabotulinumtoxinA injections in a real clinical setting.

## 2. Results

### 2.1. Patient Characteristics

A total of 216 patients underwent the first intradetrusor injection of onabotulinumtoxinA 100 IU. The median follow-up time was 10.0 months (range, 1–101 months). There were 120 (55.6%) patients who were followed up for less than 1 year, 58 (26.9%) patients for 1 to 3 years, 22 (10.2%) patients for 3 to 5 years, and 16 (7.4%) patients for more than 5 years. The mean age at the time of the procedure was 63.4 years old with 57.9% females, and 58.3% were over 65 years old. Among all patients, 76.4% had a history of taking anticholinergics, and 75.9% had a history of taking mirabegron. Six patients had an implanted SNM (InterStim, Medtronic, Minneapolis, MN, USA), but they had to be removed because there was no response to treatment.

According to a 3-day voiding diary, 58 (26.9%) patients had urgency urinary incontinence and reported a mean of 2.3 daily urgency urinary incontinence episodes at baseline (Table 1). A total of 144 patients (66.7%) underwent a urodynamic study to evaluate bladder function before onabotulinumtoxinA treatment. During filling cystometry, urodynamic detrusor overactivity was confirmed in 76.4% of the patients. The urodynamic parameters according to sex are presented in Table 2.

### 2.2. Treatment Pattern

**Figure 1:**
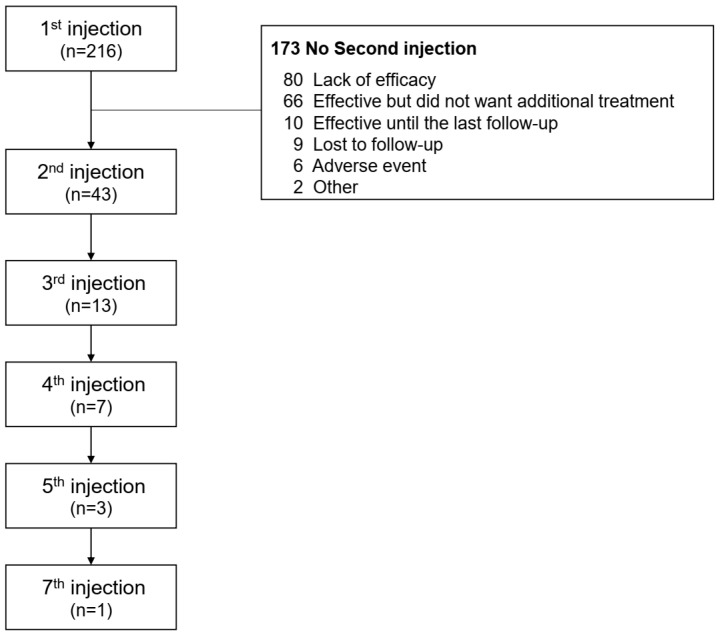
Retreatment pattern of onabotulinumtoxinA in patients (n = 216) with idiopathic OAB.

Prior to the onabotulinumtoxinA injection, 76.4% of the patients had a history of taking anticholinergics, and 75.9% had a history of taking mirabegron for at least 6 months. The proportion of patients who received a combination treatment was 30.1%. In addition, six patients underwent sacral neurostimulation, but all these patients were removed because of a lack of efficacy. Of the 216 patients who received onabotulinumtoxinA injections for the first time, 43 (19.9%) received a second treatment, and 13 (6.1%) received 3 or more injections. The reasons for not receiving the second procedure after the first injection were as follows: (1) no visit within 1 month (n = 9); (2) PVR was increased, and intermittent catheterization was initiated (n = 6); (3) lack of efficacy (n = 80); (4) patients who experienced an effect but did not want additional treatment (n = 66); and (5) efficacy was maintained until the last follow-up (n = 10) (Figure 1). Despite onabotulinumtoxinA injection, 51.4% of the patients (111/216) resumed anticholinergics or mirabegron, and the average time to resume was 2.96 months.

**Figure 2:**
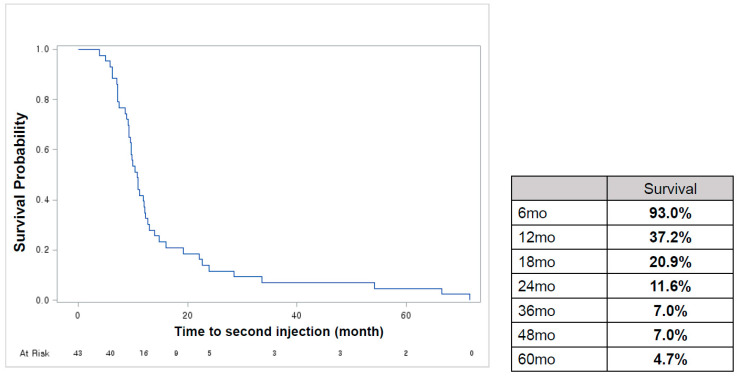
Kaplan–Meier curves for retreatment free time after the first onabotulinumtoxinA injection.

**Figure 3:**
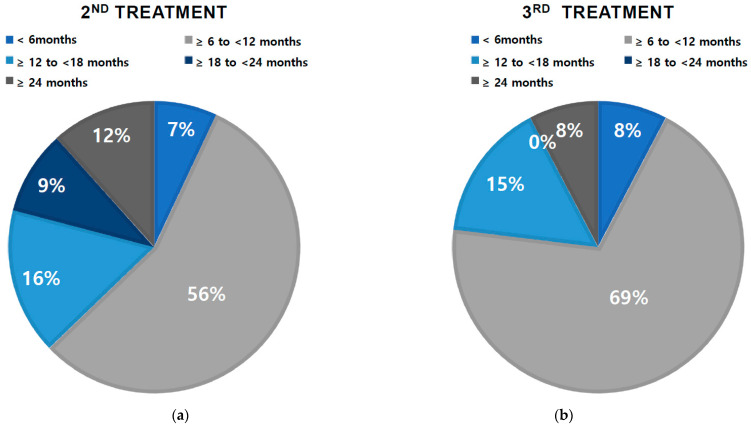
Proportion of patients retreated over time: (**a**) 2nd treatment; (**b**) 3rd treatment.

The overall median duration until the second injection was 10.7 months. Figure 2 shows the second injection-free survival curve after the first injection of onabotulinumtoxinA. Approximately 37.2% of patients did not require retreatment until 12 months after the first injection, and 11.6% did not require additional treatment even at 24 months. The median duration until the second injection was 6 months or less in 7% of patients; more than 6 months and less than 12 months in 56%; more than 12 months and less than 18 months in 16%; more than 18 months and less than 24 months in 9%; and >24 months in 12% (Figure 3a). The median duration from the second to third injection was 9.6 months. The median duration until the second injection was 6 months or less in 8% of patients; more than 6 months and less than 12 months in 69%; more than 12 months and less than 18 months in 15%; more than 18 months and less than 24 months in 0%, and >24 months in 8% (Figure 3b).

### 2.3. Outcomes

The overall patient satisfaction rate was 55.1%. At 1, 3, and 6 months after the first injection, the OABSS total and Q3 scores showed significant improvement compared with the baseline. There was a significant improvement in the mean frequency number in the voiding diary at 3 months (−3.54 ± 8.13, *p* = 0 < 0.001) and 6 months (−1.93 ± 5.42, *p* = 0.010), and a significant improvement in the mean change in nocturia (−0.53 ± 1.83, *p* = 0.010) and urgency episodes (−5.46 ± 11.97, *p* < 0.001) was observed only at 3 months. At 1, 3, and 6 months, the mean number of UUI episodes showed a decreasing trend, but this was not statistically significant (Table 3). At baseline, the mean PVR was 42.5 ± 66.4 mL. Compared with the baseline, there was a significant increase in PVR (119.4 ± 120.4 mL, *p* < 0.001) after 1 month. However, at the 3- and 6-month follow-ups, there were no significant differences observed with PVRs of 84.3 ± 92.6 mL (*p* = 0.182) and 44.3 ± 67.5 mL (*p* = 0.893), respectively.

After the second procedure, the mean changes in the OABSS total score (−1.91 ± 3.18, *p* = 0.001) and Q3 score (−0.76 ± 1.46, *p* = 0.003) at 1 month showed significant improvement. There was minimal improvement in the OABSS total score and the Q3 score at 3 and 6 months compared with the baseline, but the difference was not statistically significant. The mean numbers of frequency, urgency, and UUI in the 3-day voiding diary also decreased, but the differences were not statistically significant (Table 3).

**Figure 4:**
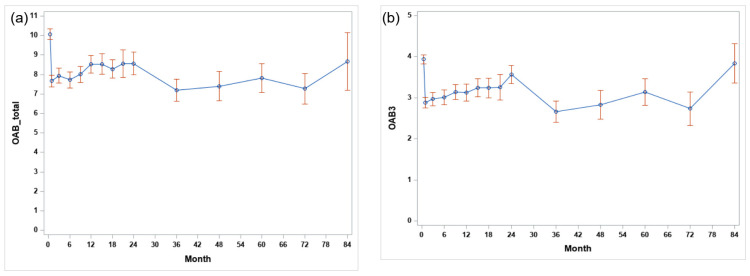
Long-term trend of (**a**) the OABSS total score and (**b**) the question 3 score for all patients after the first onabotulinumtoxinA injection.

Figure 4 shows the change in the OABSS total score and the Q3 score during the course of repeated injections and oral medication treatment according to the patients’ symptoms after the first injection. The OABSS total score improved from approximately 10 points at baseline and was maintained at 7–8 points during follow-up for 84 months. The score of Q3 for incontinence also decreased from 4 points at baseline and was maintained at an average of 2.5 to 3 points.

### 2.4. Response Rate and Predictive Factors for Response

According to the OABSS, 39.5% of the patients had a good response at 1 month, 34.4% at 3 months, and 39.7% at 6 months. In the univariate logistic regression analysis, the effects of age (OR 1.00, 95% CI; 0.95–1.03), sex (OR 1.62, 95% CI; 0.78–3.37), BCI (OR 0.99, 95% CI; 0.98–1.01), compliance (OR 1.00, 95% CI; 0.99–1.01), and refractory SNM (OR 1.02, 95% CI; 0.16–6.35) were noted. However, the presence of DO (OR 3.66, 95% CI; 1.20–11.21) and BOOI (OR 1.06; 95% CI 1.00–1.12) were found to be significantly associated with a good response (Table 4). 

The predictive factors of good responses were identified for both male and female patients. The analysis included age, presence of DO, compliance, BOOI, and BCI as independent variables (BOOI and BCI were found only in male patients). The results indicated that the presence of DO (OR 23.649, 95% CI;1.84–304.40) was a significant predictive factor for a good response in only female patients. No significant predictive factors were identified in the male patients (Table 5).

## 3. Discussion

In our study, we followed up on all patients who received onabotulinumtoxinA for refractory OAB in a real-world setting. At an average follow-up of 18 months, half of the patients were satisfied with the onabotulinumtoxinA treatment and 20% of the patients underwent two or more injections. More than half of the male patients and patients with detrusor underactivity (DU) on the UDS were included. It is also possible to combine oral drugs with onabotulinumtoxinA injections. Our results showed characteristics quite different from those of previous clinical trials. Only 20% of the patients received two or more onabotulinumtoxinA treatments. The most common reason for treatment discontinuation was a lack of effectiveness in 37% of the patients. This was significantly higher than the 5.7% reported in a prospective, long-term study [11]. However, despite the efficacy in approximately 38% of patients after the first injection, the next procedure was not performed because of a refusal of additional treatment. We believe that this result is due to the fact that many patients underwent the procedure under local anesthesia, and this is also considered a value that can only be confirmed in a real-world setting. The number of retreated patients did not reflect the efficacy of onabotulinumtoxinA injections. In another long-term study, a tolerability issue was reported as the main reason for the discontinuation of onabotulinumtoxinA injections [15]. It has been reported that intermittent catheterization is required after onabotulinumtoxinA treatment in approximately 6–7% [11,12]. In this study, only 3.3% of the patients reported that they did not consider the procedure because they needed intermittent catheterization. Patients with OAB who undergo onabotulinumtoxinA injections may experience discomfort during self-catheterization. As a result, patients may experience more discomfort and difficulty when performing self-catheterization, which can be a source of frustration and a decreased quality of life. It is important for healthcare providers to discuss this potential side effect with patients and provide appropriate education and support to manage any discomfort or difficulty with self-catheterization.

In this study, the overall duration effect was 10.7 months, which was longer than what was reported in a previous study’s 7.6 months [11]. The mean duration of the effect is an important factor in counseling patients and determining further treatment plans. Even when comparing the intervals from the prior injection to the next injection, repeated treatments did not seem to increase the effect of duration. Repeated onabotulinumtoxinA injections did not seem to increase the duration effect. This result suggests that careful consideration is required when interpreting the mean duration, given that patients can delay the treatment period even if medical staff recommend retreatment and that approximately 50% of patients resumed OAB medication treatment 3 months after onabotulinumtoxinA treatment. In one study, only 2.4% of patients reported using OAB medications 12 weeks after the first injection [16]. However, considering that the maintenance period of onabotulinumtoxinA is not long enough for 60% of patients to receive retreatment within one year, it is difficult to improve and maintain OAB symptoms using onabotulinumtoxinA treatments alone. Even after onabotulinumtoxinA treatments, appropriate medical interventions and behavioral therapies are essential to maintain the symptoms of patients whose effects are insufficient only with medication in an improved state. Studies have clearly demonstrated an increasing incidence of OAB over time as well as the persistence of symptoms that illustrate its chronic nature [18,19,20]. Thus, patients require long-term OAB management. Based on our results, we confirmed that when the OABSS was followed up in the long term, significant improvement was maintained for more than five years after the first onabotulinumtoxinA treatment (Figure 2). OAB is a disease that requires tailored or multimodal treatments based on the overall patient condition and satisfaction.

The key symptom of OAB is urgency; however, in clinical studies, improvement in urinary frequency or urgency urinary incontinence is often the criterion for efficacy. To evaluate the number of frequency or urgency urinary incontinence episodes, it is essential to collect a voiding diary for each visit. However, in real practice, instructing patients to fill out a voiding diary every time is a great burden. Therefore, in actual clinical practice, the efficacy of the current treatment is evaluated through the patient’s subjective satisfaction, response assessment, or symptom questionnaires, such as the OABSS, incontinence quality of life (I-QOL), overactive bladder-questionnaire (OAB-q), and King’s Health Questionnaire (KHQ). The success rate varied from 50 to 80%, depending on the success criteria for each study [17,20,21,22,23,24]. In this study, a decrease of three points or more in the OABSS total score was considered a good response, and approximately 40% of the patients satisfied the criteria at 6 months. We believe that the success rate was reported to be lower than that of other studies because it was strictly determined based on an OABSS reduction of three points or more. The mean change in the OABSS total score after the procedure improved significantly at 1, 3, and 6 months, and a significant improvement was confirmed in the urgency score (question number 3: How often do you have a sudden desire to urinate that is difficult to defer?). Unfortunately, in the second procedure, the treatment efficacy, similar to that of the first injection, could not be demonstrated. This is probably due to the low response rate of the voiding diary and OABSS questionnaire after the second procedure.

In clinical practice, it is important to know the treatment success rate and notify patients in advance; however, we want to know more about predicting which patients will have better treatment outcomes. If the outcome of the onabotulinumtoxinA treatment differs according to the OAB phenotype, its effectiveness can be maximized. Among the phenotypes of OAB, urodynamic DO is known to occur mainly in myogenic OAB. As onabotulinumtoxinA acts directly on the detrusor, the outcome seems to be good in idiopathic patients with DO. However, the improvement after the onabotulinumtoxinA treatment was similar based on urodynamic findings, voiding diaries, pad usage, and questionnaires regardless of the presence or absence of DO [25].

Although there are reports that the effect of onabotulinumtoxinA treatment is significantly better on female OAB patients [24], it is not well known whether the factors influencing the outcome are different between men and women [26]. We analyzed the predictive factors for outcomes separately for men and women. In our study, the presence of DO was confirmed as a predictive factor of a good response only in female patients. In a study on the effectiveness of onabotulinumtoxinA treatment in male patients, higher BOOI was reported as a negative predictive factor of treatment responses [27]. However, in our results, there were no predictive factors for a good response according to the presence of DO, age, BOOI, BCI, or degree of compliance in male patients. There was no difference in the presence of DO at baseline, which was 76.9% for males and 75.9% for females, showing different results for the factors influencing outcomes, suggesting that the causes and symptoms of OAB may be different according to sex. In men, benign prostate hyperplasia/benign prostate obstruction can also cause LUTS, with considerable overlap with the OAB symptom complex. Male OAB patients with confirmed definite BOO are not indicated for onabotulinumtoxinA treatment; however, in the absence of BOO, the outcome does not appear to have a negative effect depending on the degree of obstruction. A previous study reported that the risk of onabotulinumtoxinA treatment for detrusor overactivity with detrusor underactivity patients did not increase, but the improvement in urgency and UUI was not as good as that of OAB patients [28]. Our study did not target patients with DODU; 47 patients with a BCI < 100 were also included (data are not shown). Considering that the average BCI was 89.6, the majority of the male patients tended to have decreased bladder contractility. Although still controversial, onabotulinumtoxinA treatment is considered another option, as it does not have a negative effect on the response, and further prospective studies are required to target DODU patients.

It is difficult to avoid the limitations of this study. This was not a prospective study; the relatively poor follow-up for each patient and the collected questionnaire and voiding diaries were inconsistent. As much as possible, the analysis was performed on the OABSS and voiding diaries, which can be investigated at each visit. Nevertheless, the statistical analysis of efficacy was limited because of the small number of patients who received two or more injections.

An evaluation of urinary tract infections (UTI) was not properly performed. Several clinical studies have reported a high UTI incidence of approximately 20%. However, when the medical charts were reviewed in our study, the percentage of patients who had UTI and stopped treatment or could not be considered was not as high as expected.

## 4. Conclusions

This retrospective study aimed to evaluate the treatment patterns and long-term effectiveness of onabotulinumtoxinA injections in clinical practice. In summary, 50% of the patients were satisfied with the treatment, but only 20% received retreatment. In patients who could be followed up, the efficacy according to the OABSS questionnaire was maintained at approximately 40%, and it was well maintained even during a long-term follow-up of more than 3 years. In contrast to clinical trials, the degree of improvement and retreatment rate after onabotulinumtoxinA injections did not meet expectations.

There is a limitation in evaluating only a few variables to determine the therapeutic effect of OAB. In patients who do not respond to oral medications, onabotulinumtoxinA treatment may also be beneficial. However, it is difficult to continue OAB treatment with onabotulinumtoxinA. It is clear that OAB is a chronic disease, and maintaining long-term treatment is an important issue. During the treatment of OAB, the patient’s symptoms change over time, and physicians must provide various combinations of treatments according to the patient’s condition. In this respect, our findings provide valuable insights into the effectiveness of onabotulinumtoxinA injections in patients with refractory OAB symptoms and contribute to the optimization of treatment strategies for this debilitating condition.

## 5. Materials and Methods

This was a retrospective study conducted on OAB patients aged 18 years or older who received onabotulinumtoxinA 100 IU (Botox, Allergan, Irvine, CA, USA), administered as 20 intradetrusor injections for the first time at a single institution between April 2012 and May 2022. The study protocol was reviewed and approved by the Institutional Review Board (IRB approval no. 2023-05-027; 14 May 2022). The inclusion criteria were patients with refractory OAB symptoms despite oral medication, including antimuscarinics and mirabegron, for at least 6 months.

The preoperative 3-day voiding diary and overactive bladder symptom scores (OABSS) [29] were collected, and a urodynamic study (UDS) was selectively performed depending on the patients’ symptoms. For example, it was performed when it was necessary to exclude bladder outlet obstruction when voiding symptoms were severe in male patients or when it was necessary to confirm whether stress urinary incontinence was suspicious in female patients. For male patients, the bladder outlet obstruction index (BOOI) and bladder contractility index (BCI) were also calculated. DO was defined as a case in which involuntary detrusor contraction was confirmed using filling cystometry. In our study, we did not define DU as a quantified criterion on the UDS. DU was defined as a contraction of reduced strength and/or duration that results in prolonged bladder emptying and/or a failure to empty completely within a reasonable timespan, as referenced in the International Continence Society (ICS) standardization report [30]. It was judged by a single urologist (K-S Lee) by considering detrusor pressure, maximum flow rate, and voiding time.

All patients were followed-up at 2 weeks and 2–3 months after the injection. Subsequent visits were based on the patients’ symptoms. At each visit, we assessed patient satisfaction using a binary question of yes or no, a 3-day voiding diary, OABSS, and post-voided residual volume (PVR). PVR was assessed using an abdominal ultrasound examination. Patients could remain on their current treatment or switch to another treatment, depending on their condition. Patients showed varying response durations; therefore, we tailored the frequency of re-injection to each individual’s needs. Even if urinary symptoms improved after the injection, if it was insufficient, the patient was offered medication.

The primary outcome was the treatment pattern, including the retreatment rate and OAB medication prescription pattern. The use of anticholinergics and ß3-agonist (mirabegron) was evaluated before and after the procedure, and the time of re-administration of OAB medication after the injection was collected. The secondary outcomes were treatment satisfaction and mean changes from the baseline in daytime urinary frequency, nocturia, urgency episodes, and urgency urinary incontinence episodes per day at 1, 3, and 6 months. The minimally important difference was estimated at 3 points for the OABSS total score [31], and treatment response was defined as a decrease in the OABSS total score by 3 points or more at 1 month after the injection. At each visit, the OABSS total score and the Q3 score were evaluated to confirm the long-term patterns.

Descriptive statistics for continuous and categorical variables were presented as mean (standard deviation) and frequency (%), respectively. For each point of change compared with the baseline, a paired *t*-test or Wilcoxon signed-rank test was performed according to the normality of the change. Continuous variables between the two groups were tested using the two-sample *t*-test or Wilcoxon rank sum test according to normality, and categorical variables were tested using the chi-square test or Fisher’s exact test. To identify the predictive factors for success, multivariate logistic regression analysis was performed separately for each group of men and women. Analyses were performed using the SAS software, version 9.3 (SAS Institute Inc., Carrey, NC, USA). All inferences and descriptive *p*-values are based on two-tailed tests.

## Figures and Tables

**Table 1:** Baseline characteristics (n = 216).

Age	63.4 ± 15.5
≥65, n (%)	126 (58.3%)
≥75, n (%)	54 (25.0%)
Sex	
Male, n (%)	91 (42.1%)
Female, n (%)	125 (57.9%)
HTN, n (%)	80 (38.0%)
DM, n (%)	48 (22.2%)
Previous OAB medication history	
Anticholinergics, n (%)	165 (76.4%)
Mirabegron, n (%)	164 (75.9%)
Previous sacral neurostimulation, n (%)	6 (2.8%)
Overactive Bladder Symptom Score	
Total score, mean ± SD	10.13 ± 3.17
Q3, mean ± SD	3.95 ± 1.3
3-day voiding diary	
Frequency, mean ± SD	11.4 ± 6.8
Nocturia, mean ± SD	2.2 ± 2.0
Urgency episodes, mean ± SD	10.0 ± 9.2
Urgency urinary incontinence *, mean ± SD	2.3 ± 5.7

* Only patients (n = 58) with urgency urinary incontinence confirmed in the 3-day voiding diary before the procedure were included. DM, diabetes mellitus; HTN, hypertension; OAB, overactive bladder.

**Table 2:** Urodynamic parameters of idiopathic overactive bladder according to sex.

	Male	Female
	N = 65	N = 79
**Simple Uroflowmetry**		
Maximum flow rate (mL/s)	12.8 ± 8.3	15.3 ± 7.8
Post-voided residual (mL)	46.2 ± 71.0	39.7 ± 56.8
**Storage Phase (cystometry)**		
First sensation of bladder filling (mL)	143.6 ± 56.5	180.11 ± 92.9
First desire to void (mL)	181.1 ± 71.9	226.77 ± 99.3
Strong desire to void (mL)	260.5 ± 113.4	326.3 ± 153.6
Maximal cystometric capacity (mL)	267.6 ± 114.0	328.8 ± 126.9
Compliance	50.4 ± 41.2	70.3 ± 80.3
Detrusor overactive, n (%)	50 (76.9%)	60 (75.9%)
**Voiding Phase (pressure-flow study)**		
Detrusor underactive, n (%)	17 (26.2%)	13 (16.7%)
Bladder contractility index *	89.6 ± 40.4	-
Bladder outlet obstruction index *	22.6 ± 17.2	-

* Investigated only in men.

**Table 3:** Change from baseline in overactive bladder symptom score and 3-day voiding diary parameters.

	1st Injection	2nd Injection
**OABSS total score**		
1 month		
N	124	34
Mean change from baseline	−2.26 ± 3.38	−1.91 ± 3.18
*p*	<0.001	0.001
3 month		
N	90	16
Mean change from baseline	−2.04 ± 3.49	−1.19 ± 3.02
*p*	<0.001	0.136
6 month		
N	57	14
Mean change from baseline	−2.52 ± 3.33	−1.29 ± 4.48
*p*	<0.001	0.131
**OABSS Q3**		
1 month		
N	124	34
Mean change from baseline	−0.94 ± 1.55	−0.76 ± 1.46
*p*	<0.001	0.003
3 month		
N	90	16
Mean change from baseline	−0.97 ± 1.58	−0.69 ± 1.4
*p*	<0.001	0.069
6 month		
N	57	14
Mean change from baseline	−0.95 ± 1.49	−0.71 ± 1.86
*p*	<0.001	0.174
**3-day voiding diary parameters**		
**Frequency**		
1 month		
N	58	8
Mean change from baseline	−0.57 ± 5.33	−0.42 ± 4.19
*p*	0.865	0.852
3 month		
N	63	9
Mean change from baseline	−3.54 ± 8.13	−1.89 ± 2.37
*p*	<0.001	0.044
6 month		
N	34	4
Mean change from baseline	−1.93 ± 5.42	−4.50 ± 6.29
*p*	0.010	0.248
**Nocturia**		
1 month		
N	57	8
Mean change from baseline	0.08 ± 1.22	−0.29 ± 1.25
*p*	0.940	0.531
3 month		
N	63	9
Mean change from baseline	−0.53 ± 1.83	−0.19 ± 1.03
*p*	0.010	0.604
6 month		
N	34	4
Mean change from baseline	−0.04 ± 1.61	−0.5 ± 0.64
*p*	0.580	0.215
**Urgency**		
1 month		
N	45	6
Mean change from baseline	−0.12 ± 6.45	−1.17 ± 6.91
*p*	0.902	0.696
3 month		
N	52	9
Mean change from baseline	−5.46 ± 11.97	−3.59 ± 8.23
*p*	<0.001	0.227
6 month		
N	27	4
Mean change from baseline	−2.48 ± 7.92	−3.92 ± 5.23
*p*	0.116	0.231
**Urgency urinary incontinence**		
1 month		
N	43	6
Mean change from baseline	−0.78 ± 2.65	0.42 ± 2.88
*p*	0.096	0.738
3 month		
N	51	9
Mean change from baseline	−0.91 ± 5.11	−1.83 ± 3.51
*p*	0.098	0.156
6 month		
N	26	4
Mean change from baseline	−0.05 ± 5.22	−0.33 ± 0.47
*p*	0.348	0.252

**Table 4:** Univariate logistic regression analysis of success. A good response was defined as an OABSS total score decrease of ≥3 points at 4 weeks.

	Good Response	Poor Response	*p*	OR (95% CI)
N	49	75		
Age	62.37 ± 15.61	61.81 ± 16.6	0.851	1.00 (0.95–1.03)
Sex			0.195	1.62 (0.78–3.37)
Male	33.3%	66.7%		
Female	44.8%	55.2%		
Presence of DO			0.023	3.66 (1.20–11.21)
No	20.8%	79.2%		
Yes	50.9%	49.1%		
BOOI	35.57 ± 24.03	18.23 ± 12.41	0.041	1.06 (1.00–1.12)
BCI	82.31 ± 33.09	91.75 ± 46.78	0.510	0.99 (0.98–1.01)
Compliance	54.80 ± 47.09	65.04 ± 74.80	0.496	1.00 (0.99–1.01)
Refractory to SNM	40.0%	60.0%	0.982	1.02 (0.16–6.35)

BCI, bladder contractility index; BOOI, bladder outlet obstruction index; DO, detrusor overactivity, SNM, sacral neuromodulation.

**Table 5:** Multivariate logistic regression analysis of success (OABSS total score decreased by ≥3 points at 4 weeks) according to sex.

	Male		Female	
	*p*	OR [95% CI]	*p*	OR [95% CI]
Presence of DO	0.843	0.79 [0.08, 7.92]	0.015	23.65 [1.84, 304.40]
Age	0.862	1.00 [0.93, 1.06]	0.101	0.95 [0.894, 1.01]
BOOI *	0.063	1.07 [1.00, 1.14]	-	-
BCI *	0.833	1.00 [0.97, 1.04]	-	-
Compliance	0.710	0.99 [0.96, 1.03]	0.729	1.00 [0.99, 1.01]

BCI, bladder contractility index; BOOI, bladder outlet obstruction index; DO, detrusor overactivity. * BOOI and BCI were found only in male patients.

## Data Availability

Not applicable.
